# Effect of whey vs. soy protein supplementation on recovery kinetics following speed endurance training in competitive male soccer players: a randomized controlled trial

**DOI:** 10.1186/s12970-021-00420-w

**Published:** 2021-03-16

**Authors:** Savvas Kritikos, Konstantinos Papanikolaou, Dimitrios Draganidis, Athanasios Poulios, Kalliopi Georgakouli, Panagiotis Tsimeas, Theofanis Tzatzakis, Dimitrios Batsilas, Alexios Batrakoulis, Chariklia K. Deli, Athanasios Chatzinikolaou, Magni Mohr, Athanasios Z. Jamurtas, Ioannis G. Fatouros

**Affiliations:** 1 Department of Physical Education and Sport Science, University of Thessaly, Karies, 42100 Trikala, Greece; 2 Department of Nutrition and Dietetics, University of Thessaly, Argonafton 1, 42132 Trikala, Greece; 3 Department of Physical Education and Sport Sciences, Democritus University of Thrace, 69100 Komotini, Greece; 4 Department of Sports Science and Clinical Biomechanics, SDU Sport and Health Sciences Cluster (SHSC), Faculty of Health Sciences, University of Southern Denmark, Odense, Denmark; 5 Centre of Health Science, Faculty of Health Sciences, University of the Faroe Islands, Tórshavn, Faroe Islands

**Keywords:** Intensified training, Recovery, Exercise-induced muscle damage, Protein ingestion, Performance

## Abstract

**Background:**

Soccer-specific speed-endurance training induces short-term neuromuscular fatigue and performance deterioration over a 72-h recovery period, associated with elevated markers of exercise-induced muscle damage. We compared the effects of whey vs. soy protein supplementation on field activity, performance, muscle damage and redox responses following speed-endurance training in soccer players.

**Methods:**

Ten well-trained, male soccer players completed three speed-endurance training trials, receiving whey protein (WP), soy protein (SP) or an isoenergetic placebo (PL; maltodextrin) according to a randomized, double-blind, crossover, repeated-measures design. A pre-loading period was applied in each trial during which protein supplementation was individually adjusted to reach a total protein intake of 1.5 g/kg/day, whereas in PL protein intake was adjusted at 0.8–1 g/kg/day. Following pre-loading, two speed-endurance training sessions (1 and 2) were performed 1 day apart, over a 3-day experimental period. During each session, field activity and heart rate were continuously monitored using global positioning system and heart rate monitors, respectively. Performance (isokinetic strength of knee extensors and flexors, maximal voluntary isometric contraction, speed, repeated sprint ability, countermovement jump), muscle damage (delayed-onset of muscle soreness, creatine kinase activity) and redox status (glutathione, total antioxidant capacity, protein carbonyls) were evaluated at baseline (pre), following pre-loading (post-load), and during recovery from speed-endurance training.

**Results:**

High-intensity and high-speed running decreased (*P* ≤ 0.05) during speed-endurance training in all trials, but WP and SP mitigated this response. Isokinetic strength, maximal voluntary isometric contraction, 30-m speed, repeated sprint ability and countermovement jump performance were similarly deteriorated during recovery following speed-endurance training in all trials (*P* ≤ 0.05). 10 m speed was impaired at 24 h only in PL. Delayed-onset of muscle soreness, creatine kinase, total antioxidant capacity and protein carbonyls increased and glutathione decreased equally among trials following speed-endurance training (*P* ≤ 0.05), with SP inducing a faster recovery of protein carbonyls only at 48 h (*P* ≤ 0.05) compared to WP and PL.

**Conclusions:**

In conclusion, increasing daily protein intake to 1.5 g/kg through ingestion of either whey or soy protein supplements mitigates field performance deterioration during successive speed-endurance training sessions without affecting exercise-induced muscle damage and redox status markers.

**Trial registration:**

Name of the registry: clinicaltrials.gov. Trial registration: NCT03753321. Date of registration: 12/10/2018.

**Supplementary Information:**

The online version contains supplementary material available at 10.1186/s12970-021-00420-w.

## Background

Soccer engages both oxidative and non-oxidative energy pathways [1] and therefore, high-intensity drills targeting these energy pathways are considered essential in competitive soccer training. Speed-endurance training is incorporated during the in-season microcycle to improve players’ ability to perform repeated maximal sprints and develop fatigue resistance [2, 3]. Speed-endurance training includes brief (10–40 s) repeated sprints that stimulate the phosphagen and glycolytic pathways [4], interspersed by periods of recovery (2–4 min) using a work-to-rest ratio of ≥1:5 [5].

Speed-endurance training incorporates activities with a strong eccentric component (i.e. high-speed running, accelerations, decelerations and changes of direction) which may promote exercise-induced muscle damage and results in prolonged performance deteriorations. In fact, a previous study [6] revealed that speed-endurance training induces short-term neuromuscular fatigue and prolonged elevation of exercise-induced muscle damage markers accompanied by performance deterioration, for as long as 72 h during recovery.

The potential of protein supplements to enhance performance recovery in team sports by alleviating exercise-induced muscle damage has been examined and reviewed [7, 8]. Although, the effectiveness of protein supplementation remains currently equivocal, potentially due to large variability of study designs and exercise-induced muscle damage-related markers examined [7, 8], there are indications that animal- and plant-based proteins such as whey (WP) and soy (SP) may elicit a beneficial effect on muscle function and physical performance following damaging exercise [9, 10]. Both protein types are considered fast-digested (as compared to other types of protein such as casein) [11], though WP is considered to be superior to SP in stimulating muscle protein synthesis due to a greater essential amino acids and leucine content and a greater amino-acid bioavailability [11]. Furthermore, WP and SP are both rich in cysteine, an amino acid that serves as a substrate for intracellular glutathione synthesis, and thus may exert antioxidant properties [12]. However, the processing of animal-based proteins to produce nutritional supplements is associated with higher economic cost and environmental burden (i.e. greater greenhouse gas emissions, more water and land is required), that necessitates the consumption of more sustainable and environmental friendly protein sources, such as plant-based proteins [13].

To the best of our knowledge, there is limited or inconsistent evidence with respect to the role of milk- and soy-based protein supplementation in enhancing performance recovery and skeletal muscle healing following team-sport activity [8], while the extent to which the type of protein administered impacts these responses, is currently unexplored. Therefore, the aim of this study was to investigate whether protein supplementation can alleviate exercise-induced muscle damage and blood oxidative stress responses and to enhance performance recovery following speed-endurance training in soccer players. Furthermore, we sought to determine the effect of WP versus SP. Given the greater essential amino acid profile and leucine availability in WP compared to SP, we hypothesized that WP would be superior to SP in promoting recovery.

## Methods

### Study design

**Fig. 1:**
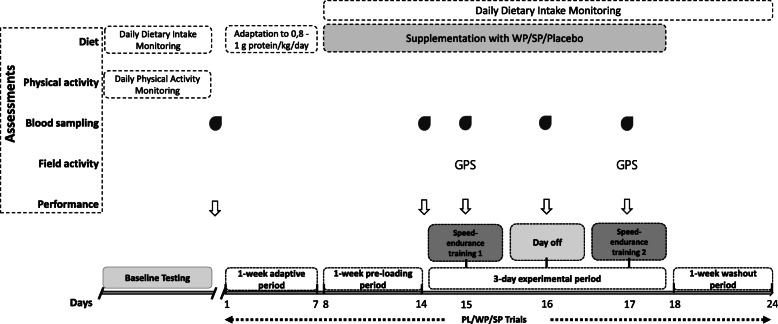
The experimental design of the study. WP: whey protein; SP: soy protein; PL: placebo

The experimental flowchart of the study is shown in Fig. 1. A randomized, three-trial, (placebo vs. WP vs. SP), cross-over, double-blind, repeated measures design was implemented. Initially, all participants underwent baseline testing, including assessment of their anthropometric profile (body mass, height), body composition, resting metabolic rate, daily dietary intake, habitual physical activity, physical conditioning level (maximal oxygen consumption, Yo-Yo intermittent endurance level 2, Yo-Yo intermittent recovery level 2) and technical skills (creative speed test and short dribbling test), over a 7-day period. Thereafter, they participated randomly (using an online semi-automated software) in three trials: (i) Placebo (PL), (ii) Whey Protein (WP) and (iii) Soy Protein (SP) supplementation.

Each trial included (i) an initial 1-week adaptive period (days 1–7), during which participants received individualized dietary plans providing them a daily protein intake of 0.8–1 g of protein/kg/day. This period included only very light-load soccer practice (consisted mainly of familiarization with the experimental training protocol) of limited duration (< 30 min per session) while participants were asked to abstain from any moderate-to-vigorous daily physical activity. (ii) a subsequent a 7-day pre-loading period (days 8–14), during which participants consumed the protein or placebo supplement (depending on the trial) on a daily basis. (iii) a 3-day experimental period, consisted of two speed-endurance training sessions performed 48 h apart (day 15 and day 17, respectively) with participants following the same dietary plan and supplementation protocol (protein supplement or placebo) as in the pre-loading phase. During the experimental period, participants were engaged only very light training of limited duration and avoided any moderate-to-vigorous physical activity as in the adaptive period. In-between the two speed-endurance training sessions (day 16), no training was performed. (iv) a 7-day washout period, during which participants were engaged only in daily very light training and followed the balanced dietary plan of the adaptive period. The washout period aimed at alleviating any inflammatory and muscle damaging effect induced by the previous trial prior to participation of the next one. The second training session was added to assess the effectiveness of the experimental treatments on the recovery kinetics of field performance.

Performance assessment (i.e. delayed-onset of muscle soreness, isokinetic strength, maximal voluntary isometric contraction, 10 m and 30 m speed, countermovement jump, repeated sprint ability) and resting blood sampling for the determination of biochemical indices related to exercise-induced muscle damage and redox status (i.e. creatine kinase, glutathione, total antioxidant capacity and protein carbonyls) were performed before each trial (pre; prior to the adaptive period) and at the end of pre-loading (post-load). By including the assessment at post-load we aimed at determining any potential alteration on dependent variables induced by WP vs SP supplementation per se. To assess the ability of WP and SP in enhancing recovery following speed-endurance training session 1, isokinetic strength, 10 m and 30 m speed, countermovement jump, repeated sprint ability were assessed at 24 h, maximal voluntary isometric contraction was tested at 1, 2, 3, 24 and 48 h (day 17, prior to speed-endurance training 2), while blood sampling and assessment of the delayed-onset of muscle soreness were performed at 24 and 48 hous (day 17, prior to speed-endurance training 2). Additional blood samples were drawn before and immediately after each speed-endurance training session for the determination of blood lactate concentration. During speed-endurance training sessions, field locomotor activity and heart rate responses were continuously monitored using global positioning system instrumentation and heart rate monitors.

Performance testing and blood sampling at all time-points were performed in the fed state and at the same time of day in each trial to prevent circadian rhythm variations. Similarly, speed-endurance training sessions were performed at the same time of day in each trial (17:00–18:00) under comparable environmental conditions (20–25 °C, ~ 60% humidity). Before each session, a standard breakfast and meal was consumed by all participants, and during each session they were allowed to consume only water ad libitum*.* Dietary intake was monitored daily during each trial.

### Participants

A power analysis (effect size of 0.3, power of 0.80, probability error of 0.05, 2-tailed) for main variables and within-between factors repeated-measures analyses of variance, indicated a sample size of 8–10 participants. Accordingly, 12 soccer players were assessed for eligibility and 10 of them were finally included in the study (see Additional file 1). Participation was secured if volunteers (1) participated at a competitive level for ≥4 years (≥5 training sessions/week, ≥1 match/week), (2) were illness- and injury-free, (3) abstained (≥6 months before the study) from consumption of ergogenic supplements or medication and (4) were non-smokers. Participants’ characteristics at baseline are shown in Table 1. Experimental procedures were applied in alignment with the Declaration of Helsinki, as revised in 2013. Participants signed an informed consent after they had being informed about the goals of the study and its associated risks and benefits. The study was approved by the University of Thessaly Institutional Ethics Committee (1412/1–5/3–10/2018).

**Table 1:** Participants’ characteristics at baseline (*n* = 10)

Age (years)	21 ± 1.5
Weight (kg)	79.3 ± 6.8
Height (m)	1.80 ± 0.1
BMI (kg/m^2^)	24.6 ± 1.2
Body fat (%)	19 ± 6.7
Lean body mass (kg)	59.9 ± 6.5
RMR (kcals/day)	2021.6 ± 232.3
Habitual energy expenditure (kcals/day)^a^	152.9 ± 55.7
VO_2_max (ml/kg/min)	59.0 ± 4.8
Yo-Yo IE2 (m)	2192 ± 308.6
Yo-Yo IR2 (m)	1230 ± 162.3
Short dribbling test (sec)	13.2 ± 0.9
Creative speed test (sec)	17.8 ± 0.7

^a^Denotes energy expenditure at baseline. *BMI* body mass index, *RMR* resting metabolic rate, *VO*_*2*_*max* maximal oxygen consumption, *IE* intermittent endurance, *IR* intermittent recovery

### Speed-endurance training

Speed-endurance training (~ 60 min) was performed on natural grass and incorporated 1 set of 8 (30-s each) maximum-intensity repetitions with a passive recovery of 2.5 min (work-to-rest ratio of 1:5) and utilized a soccer-specific drill, as described [6]. The net exercise time of the protocol was ~ 4 min. Players were verbally encouraged during each repetition to perform at maximum intensity. In each session, speed-endurance training was preceded by a standard 15-min warm-up (shuttle running, dynamic stretching, agility drills) and followed by a 15-min cool-down (light-intensity running, passive stretching).

### Dietary intervention and supplementation protocol

Participants’ daily energy and macronutrient intake were individually adjusted during the course of each trial based on dietary analysis, resting metabolic rate and physical activity-related energy expenditure (performed at baseline). Furthermore, given the reduced energy expenditure and training load of our participants throughout the study and the limited net exercise time of the two experimental exercise protocol, individual dietary plans were adapted to the current recommendations of the International Society of Sports Nutrition for individuals participating in fitness programs of low intensity and low volume [14] and as such total energy and macronutrient intake were much below the respective amounts recommend for soccer players during the in-season [15] (see Table 2). Accordingly, each participant received 28–29 kcals/kg/day (2190–2261 kcals per day), 3–3.6 g of carbohydrate/kg/day (43–52% of total energy) and 1.05–1.13 g of fat/kg/day (35–36% of total energy). Daily protein intake was set to 0.8–1 g of protein/kg during the adaptive period in an attempt to equalize the relative amount of protein received by participants prior to supplementation. To meet the study’s goal for increased protein consumption during pre-loading and the subsequent 3-day experimental period in WP and SP trials (in PL trial daily protein intake remained at 0.8–1 g/kg/day), protein intake was increased to 1.5 g/kg/day through supplementation, as recommended for individuals engaged in moderate amounts of high-intensity training [14].

**Table 2:** Participants’ dietary intake and antioxidant profile during the adaptive and experimental period

	Placebo			Whey			Soy		
	Diet	Supplement	Total	Diet	Supplement	Total	Diet	Supplement	Total
**Daily energy intake**									
**kcal/day**	**1971.9 ± 446.4**	**236.0 ± 0.0**	**2207.9 ± 446.4**	**1957.6 ± 539.2**	**231.9 ± 0.00**	**2189.5 ± 539.2**	**2028.1 ± 376.6**	**233.1 ± 0.0**	**2261.2 ± 376.6**
**Carbohydrate intake**									
**g/kg body mass**	**2.87 ± 0.6**	**0.74 ± 0.06**	**3.62 ± 0.6**	**2.98 ± 0.8**	**0.0 ± 0.0**	**2.98 ± 0.8**	**3.05 ± 0.6**	**0.04 ± 0.0**	**3.09 ± 0.6**
**% of total energy intake**	**46.2 ± 3.4**	**11.0 ± 2.11**	**52.0 ± 3.4**	**48.3 ± 3.0**	**0.0 ± 0.0**	**43.2 ± 3.0**	**47.7 ± 3.2**	**0.50 ± 0.0**	**43.4 ± 3.2**
**Protein intake**									
**g/kg body mass**	**0.81 ± 0.05**	**0.0 ± 0.0**	**0.81 ± 0.05**	**0.83 ± 0.2**	**0.69 ± 0.06**	**1.52 ± 0.2**^**a**^	**0.82 ± 0.1**	**0.68 ± 0.06**	**1.51 ± 0.2**^**b**^
**% of total energy intake**	**13.0 ± 2.50**	**0.0 ± 0.0**	**11.6 ± 2.50**	**13.4 ± 1.4**	**10.5 ± 2.50**	**22.0 ± 2.9**^**a**^	**12.8 ± 1.0**	**9.8 ± 1.8**	**21.2 ± 1.5**^**b**^
**Fat intake**									
**g/kg body mass**	**1.13 ± 0.3**	**0.0 ± 0.0**	**1.13 ± 0.3**	**1.05 ± 0.2**	**0.01 ± 0.00**	**1.07 ± 0.2**	**1.12 ± 0.2**	**0.00 ± 0.00**	**1.12 ± 0.2**
**% of total energy intake**	**40.8 ± 4.4**	**0.0 ± 0.0**	**36.4 ± 4.4**	**38.3 ± 3.7**	**0.6 ± 0.1**	**34.8 ± 3.7**	**39.5 ± 3.3**	**0.12 ± 0.01**	**35.4 ± 3.4**
**Selenium (μg/day)** ^**c**^	**50.1 ± 11.2**								
**Zinc (mg/day)** ^**c**^	**11.5 ± 1.90**								
**Vitamin C (mg/day)** ^**c**^	**117.0 ± 17.20**								
**Vitamin E (mg/day, α-TE)** ^**c**^	**8.4 ± 2.0**								

^**a**^Significant difference between Whey and Placebo at *P* < 0.05. ^**b**^Significant difference between Soy and Placebo at *P* < 0.05. ^**c**^Daily intake of these compounds was comparable among trials

Supplementation during the three trials (i.e. throughout pre-loading and the 3-day experimental period) included WP isolate (Instantized BiPRO® I.P., Davisco Foods International, INC, Minnesota, USA - WP: 91 g; carbohydrates: 0 g; fat: 1.3 g; 380 kcal per 100 g), SP isolate (Soy Protein Isolate, My Protein, UK – SP: 93 g; carbohydrates: 1 g; fat: 3 g; 379 kcal per 100 g) or isoenergetic placebo (Maltodextrin, My Protein, UK) in a random order. Specifically, during pre-loading and the day in-between training sessions (day 16), drinks were consumed in a single dose with breakfast, whereas on training days (days 15 and 17), drinks were administered immediately post-training as a single bolus. The protein content in each protein drink was individually adjusted, based on each participant’s dietary protein intake, to account for a total daily protein consumption of 1.5 g/kg/day. All drinks were isovolumetric (~ 500 ml), consumed with water and flavored with ~ 10 drops of non-caloric sweetener (Flavdrops chocolate, My Protein, UK) to make the contents indistinguishable and non-transparent. Participants were asked 9 times each (once/day) if they knew what was the ingested solution. Out of a total of 270 responses, 129 times answered “I do not know”, 85 times answered incorrectly and 56 times answered correctly (possibly due to chance). Hence, we believe that participants were well-blinded. During trials, participants adhered to a daily nutrition plan that included three meals and two snacks. Daily protein intake was derived from dairy, eggs, poultry, meat and nuts.

### Diet monitoring and analysis

A registered dietitian instructed participants how to record food/fluid servings and their daily dietary intake. Then, they completed 7-day diet recalls to evaluate their macronutrient and energy intake using a nutritional software (Science Fit Diet 200A, Science Technologies, Athens, Greece) as previously described [16, 17]. Participants’ daily dietary intake was also monitored and analyzed during all trials and the wash-out periods to ensure that there were no deviations from the prescribed diet.

### Field activity

Field activity and internal load during speed-endurance training was monitored using high time resolution global positioning system equipped with heart rate monitors (10-Hz GPS, 200-Hz triaxial accelerometry; Polar Team Pro, Polar Electro, Kempele, Finland) as previously described [18]. Field activity was classified as total distance; average and maximum speed; high-intensity running (distance covered at speeds 14–21 km/h); high-speed running (distance covered at speeds > 21 km/h); intense accelerations and decelerations counts (> 2 m/s^2^). Internal load was expressed as maximum and average heart rate.

### Descriptives

Body mass and height were measured on a beam balance with a stadiometer (Beam Balance Stadiometer, SECA, Vogel & Halke, Hamburg, Germany) as described [17]. Dual-energy X-ray absorptiometry (DXA, GE Healthcare, Lunar DPX-NT) was utilized for body composition assessment as previously published [19]. Open-circuit spirometry with an automated online pulmonary gas exchange system (Vmax Encore 29, BEBJO296, Yorba Linda, CA, USA) was used for the assessment of maximal oxygen consumption, via breath-by-breath analysis during a graded exercise testing on a treadmill (Stex 8025 T, Korea) as previously described [18]. For resting metabolic rate measurement, resting VO_2_/CO_2_ values were measured in the morning (07.00–09.00) after an overnight fast using an open-circuit indirect calorimeter with a ventilated hood system (Vmax Encore 29, BEBJO296, Yorba Linda, CA, USA) and the 24 h-resting metabolic rate was calculated as previously described [17]. Physical activity levels were monitored over a 7-day period via 3-axial accelerometers (ActiGraph GT3X+, Pensacola, FL, USA) as described [16]. Soccer-specific conditioning was measured using the Yo-Yo intermittent endurance level 2 and Yo-Yo intermittent recovery level 2 tests as previously described [20]. Participants’ level of technical ability was determined using the creative speed and short dribbling tests as described elsewhere [6].

### Performance

Maximal voluntary isometric contraction as well as concentric and eccentric isokinetic peak torque of the knee extensors and knee flexors (at 60°/s) were measured on an isokinetic dynamometer (Cybex Norm 770; Cybex, Ronkonkoma, NY), both in dominant and non-dominant limb, as described [21]. Countermovement jump was measured on an Ergojump contact platform (NewTest Ltd., Kiviharjuntie, Finland) as described [22]. Sprint time over 10- and 30-m, and repeated sprint ability fatigue index assessments were performed using photocells [18]. Delayed-onset of muscle soreness of the knee extensors and knee flexors of the dominat limb was assessed by palpation [21].

### Blood sampling and assays

Fasting blood samples were collected by venipuncture using a disposable 20-gauge needle from an antecubital arm vein with the participants seated. For serum separation (to measure creatine kinase and total antioxidant capacity), samples were collected in tubes containing SST-Gel/clot activator and allowed to clot at room temperature for 30 min before centrifuged (1370 g, 4 °C, 10 min). For plasma separation (to measure protein carbonyls), samples were collected in tubes containing ethylenediaminetetraacetic acid and centrifuged immediately (1370 g, 4 °C, 10 min). Red blood cell lysates were prepared (to measure hemoglobin and glutathione) after lysis of packed erythrocytes following plasma separation. Samples were stored at − 80 °C in multiple aliquots until assayed. Samples were protected from light and auto-oxidation and thawed once before measured in duplicate.

Blood lactate concentration was assessed using a hand portable analyzer (Lactate Plus; Nova Biomedical, Waltham, MA) as described [18]. Creatine kinase was measured using an automated Clinical Chemistry Analyzer Z1145 (P. Zafiropoulos S.A., Athens, Greece) with commercially available kits (P. Zafiropoulos S.A.) as previously described [20]. Glutathione, total antioxidant capacity and protein carbonyls were analyzed spectrophotometrically as previously described [18]. Spectrophotometric assays were performed on a Hitachi 2001 UV/VIS (Hitachi Instruments Inc., U.S.). The inter- and intra-assay coefficients ranged from 1.5 to 7.2% for all assays.

### Statistical analyses

Data are presented as means±SD. Normality was examined using the Shapiro-Wilk test. Performance, muscle damage and redox status variables were analyzed using a two-way (condition vs time), repeated-measures ANOVA with planned contrasts on different time points. When a significant interaction was detected, a Bonferonni test was applied for post-hoc analysis. Percentage change from speed-endurance training 1 to 2 (Δchange) of field activity data in each trial were analyzed using a one-way ANOVA with a Bonferroni correction for multiple comparisons. For all dependent variables, effect sizes and confidence intervals were calculated according to the corrected for bias Hedge’s method (see Additional file 2). Effect sizes were considered as none, small, medium-sized and large for values 0.00–0.19, 0.20–0.49, 0.50–0.79 and ≥ 0.8, respectively. The IBM SPSS Statistics for Windows was used for analyses (version 20; IBM Corp., Armonk, NY) with significance accepted at *P* ≤ 0.05.

## Results

Values for all dependent variables prior to the adaptive period (Pre) were comparable among trials, indicating that the 1-week wash-out period was sufficient for eliminating any exercise-induced muscle damage and inflammatory effects from the previous trial. Moreover, none of the dependent variables changed significantly following the pre-loading phase (post-load) in any trial. Dietary analysis showed that participants followed a standard diet throughout the study, providing them with 2189,5–2261,2 kcals/day (Table 2). Their daily energy expenditure, based on resting metabolic rate (measured at baseline) and habitual physical activity (Table 1), was estimated to 2174 kcals/day whereas, the speed endurance training sessions induced an additional energy expenditure of 95–107 kcals (see Additional file 3). No adverse side effects were reported in response to protein and placebo supplementation.

### Field activity and internal load during speed-endurance training

**Fig. 2:**
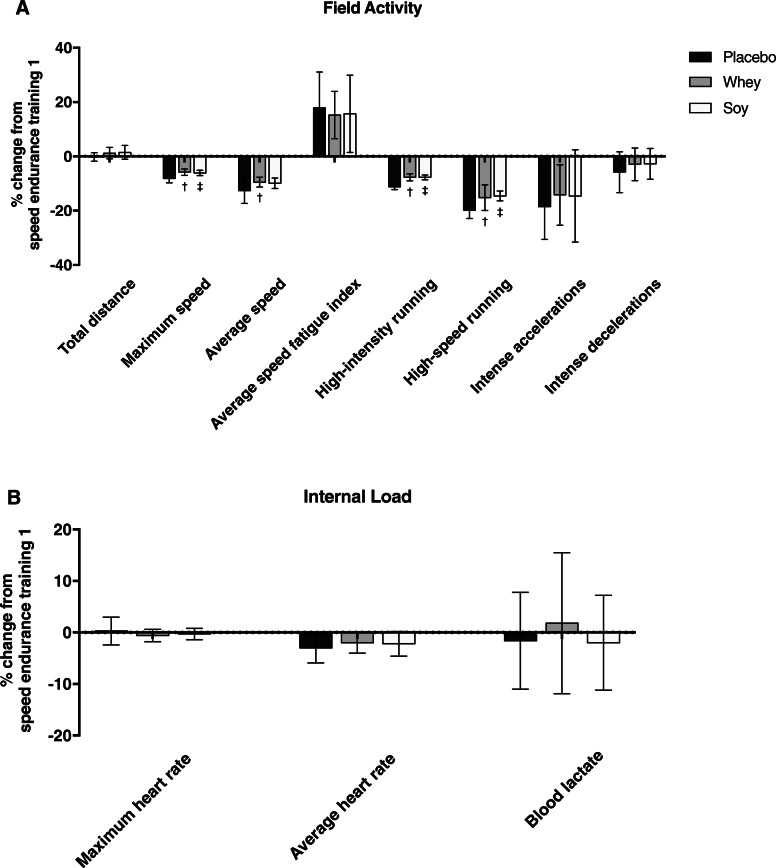
Changes (percentage changes from speed-endurance training session 1) in field activity (**a**) and internal load (**b**) indices during speed-endurance training session 2. ^†^denotes significant difference between whey and placebo trial at *P* < 0.05. ^‡^ denotes significant difference between soy and placebo trial at *P* < 0.05

Figure 2 illustrates changes in field activity (Fig. 2a) and internal load (Fig. 2b) during speed-endurance training session 2 (see also Additional file 3). Total distance covered in speed-endurance training sessions was comparable among trials. Maximum speed was lower in speed-endurance training 2 compared to speed-endurance training 1 in all trials (PL: -8.1%, *P* < 0.001; WP: -5.8%, *P* < 0.001; SP: -6.1% *P* < 0.001), with WP and SP inducing a decline of smaller magnitude compared to PL by 2.3% (*P* < 0.05) and 2% (*P* < 0.05), respectively. Likewise, average speed decreased during speed-endurance training 2, as compared to speed-endurance training 1, in all trials (PL: -12.6%, *P* < 0.001; WP: -9.5%, *P* < 0.001; SP: -9.9%, *P* < 0.001), with WP inducing a higher average speed during speed-endurance training 2 compared to PL (by 5%, *P* < 0.05). Average speed fatigue index (%) was higher during speed-endurance training 2 in all trials (PL: + 17.9%, *P* < 0.001; WP: + 15.2%, *P* < 0.001; SP: + 15.7%, *P* < 0.05) without any effect of protein supplementation. High-intensity running decreased significantly during speed-endurance training 2 in all trials (PL: -11.2%, *P* < 0.001; WP: -7.7%, *P* < 0.001; SP: -7.7%, *P* < 0.001), with WP and SP significantly mitigating this response (*P* < 0.001). Similarly, high-speed running was lower in speed-endurance training 2 compared to speed-endurance training 1 in all trials (PL: -19.8%, *P* < 0.001; WP: -15.2%, *P* < 0.001; SP: -14.6%, *P* < 0.001) with WP and SP inducing a drop of smaller magnitude compared to PL by 4.6% (*P* < 0.001) and 5.2% (*P* < 0.001), respectively. Intense accelerations decreased during speed-endurance training 2 in all trials (PL: -18.5%, *P* < 0.001; WP: -14.2%, *P* < 0.05; SP: -14.6%, *P =* 0.001), without any effect or protein supplementation, while intense decelerations decreased significantly during speed-endurance training 2 only in PL (by 5.8%, *P* < 0.05). Maximum heart rate was similar between speed-endurance training 1 and 2 in all trials, whereas average heart rate was lower in speed-endurance training 2 compared to speed-endurance training 1 in all trials (PL: -3%, *P* < 0.001; WP: -2%, *P* < 0.05; SP: -2.2%, *P* < 0.05). Blood lactate concentration increased similarly following speed-endurance training 1 and 2, in all trials (10- to 12-fold rise, *P* < 0.001), without any effect of protein supplementation. The mean energy expenditure during speed-endurance training sessions was 95–108 kcals and it was comparable among trials (see Additional file 3).

### Performance

Concentric and eccentric strength of knee extensors and knee flexors of the dominant and non-dominant limb decreased similarly in all trials at 24 h after speed-endurance training 1 (*P* < 0.05), as compared to pre-exercise levels (Table 3). Maximal voluntary isometric contraction decreased (compared to pre) only in knee extensors of the non-dominant limb 1 h (by 12–13%, *P* < 0.001) and 2 h (by 7–7.6%, *P* < 0.05) following speed-endurance training 1 in all trials, without any effect of protein supplementation (see Additional file 4). Although there was observed a similar decline of maximal voluntary isometric contraction in knee extensors of the dominant limb in all trials at 1 h (by 11.8–13%) and 2 h (by 7.3–8.4%) after speed-endurance training 1, it didn’t reach statistical significance probably because of the large standard deviation.

**Table 3:** Changes in performance indices

	Placebo			Whey			Soy		
	Pre	Post-Load	24 h	Pre	Post-Load	24 h	Pre	Post-Load	24 h
CON-KE-DL (Nm)	258.4 ± 25.1	256.2 ± 25.8	220.8 ± 26.2^a^	259.0 ± 33.4	256.3 ± 26.8	230.7 ± 32.4^b^	255.8 ± 30.0	258.3 ± 23.0	227.3 ± 33.9^c^
CON-KE-NDL (Nm)	257.8 ± 33.5	255.1 ± 33.1	221.6 ± 31.5^a^	256.9 ± 28.7	258.7 ± 26.2	227.3 ± 31.1^b^	257.8 ± 31.2	256.2 ± 24.7	229.4 ± 22.7^c^
CON-KF-DL (Nm)	157.0 ± 22.8	156.4 ± 22.6	142.1 ± 22.0^a^	158.2 ± 21.7	156.9 ± 23.5	144.0 ± 21.1^b^	156.2 ± 23.9	155.4 ± 22.6	141.8 ± 22.4^c^
CON-KF-NDL (Nm)	155.1 ± 23.2	155.9 ± 22.9	140.6 ± 26.2^a^	156.7 ± 22.4	155.5 ± 23.8	142.4 ± 21.5^b^	154.9 ± 22.8	154.0 ± 23.5	141.6 ± 22.1^c^
ECC-KE-DL (Nm)	330.5 ± 73.5	333.9 ± 74.1	281.7 ± 81.6^a^	332.6 ± 72.7	331.4 ± 72.9	289.9 ± 80.6^b^	330.6 ± 72.6	332.8 ± 74	291.8 ± 65.4^c^
ECC-KE-NDL (Nm)	329.2 ± 47.8	328.3 ± 48.0	284.9 ± 44.4^a^	329.7 ± 53.8	328.6 ± 51.4	288.4 ± 54.5^b^	328.9 ± 52.0	329.9 ± 48.8	289.2 ± 49.5^c^
ECC-KF-DL (Nm)	193.3 ± 31.3	192.0 ± 32.0	154.1 ± 31.1^a^	195.8 ± 24.4	197.4 ± 25.7	167.0 ± 28.9^b^	196.5 ± 25.1	194.7 ± 20.0	164.8 ± 23.8^c^
ECC-KF-NDL (Nm)	192.7 ± 18.7	193.5 ± 23.4	156.4 ± 27.8^a^	194.2 ± 21.1	193.4 ± 26.2	168.2 ± 20.1^b^	195.4 ± 22.1	194.0 ± 19.6	166.5 ± 26.3^c^
10 m sprint time (sec)	1.908 ± 0.076	1.938 ± 0.099	2.020 ± 0.125^a^	1.934 ± 0.068	1.942 ± 0.069	1.984 ± 0.090	1.896 ± 0.076	1.931 ± 0.081	1.951 ± 0.136
30 m sprint time (sec)	4.580 ± 0.140	4.565 ± 0.161	4.818 ± 0.147^a^	4.587 ± 0.172	4.574 ± 0.179	4.735 ± 0.178^b^	4.567 ± 0.150	4.591 ± 0.130	4.743 ± 0.145^c^
RSA fatigue index (%)	6.39 ± 1.23	6.43 ± 1.16	7.65 ± 1.57^a^	6.45 ± 1.07	6.43 ± 1.02	7.32 ± 1.27^b^	6.41 ± 0.96	6.44 ± 0.73	7.43 ± 0.80^c^
CMJ (cm)	47.1 ± 3.4	47.6 ± 4.3	43.8 ± 3.6^a^	47.3 ± 3.1	47.0 ± 3.0	44.9 ± 2.5^b^	46.9 ± 3.2	46.5 ± 3.0	44.3 ± 2.5^c^

*CON* concentric, *ECC* eccentric, *KE* knee extensors, *KF* knee flexors, *DL* dominant limb, *NDL* non-dominant limb, *RSA* repeated sprint ability, *CMJ* countermovement jump. Note: Data are presented as means ± SD. ^a^ denotes a significant difference with pre in placebo at *P* < 0.05. ^b^ denotes a significant difference with pre in whey at *P* < 0.05. ^c^ denotes a significant difference with pre in soy at *P* < 0.05

Speed over 10-m was substantially lower than baseline at 24 h after speed-endurance training 1 only in PL by ~ 6% (*P* = 0.03), whereas 30-m speed was similarly decreased at 24 h after speed-endurance training 1 in PL, WP and SP by 5.2% (*P* < 0.001), 3.2% (*P* < 0.001) and 3.9% (*P* < 0.001), respectively, as compared to pre, without any effect of protein supplementation (Table 3). Repeated sprint ability fatigue index increased (PL: + 20%, *P* < 0.001; WP: + 13%, *P* = < 0.05; SP: + 16%, *P* = 0.001) and countermovement jump height decreased (PL: -7%, *P* < 0.001; WP: -5%, *P* < 0.05; SP: -5%, *P* = 0.001) similarly in all trials at 24 h after speed-endurance production training 1, compared to their baseline values (Table 3).

### Exercise-induced muscle damage and blood redox status

**Fig. 3:**
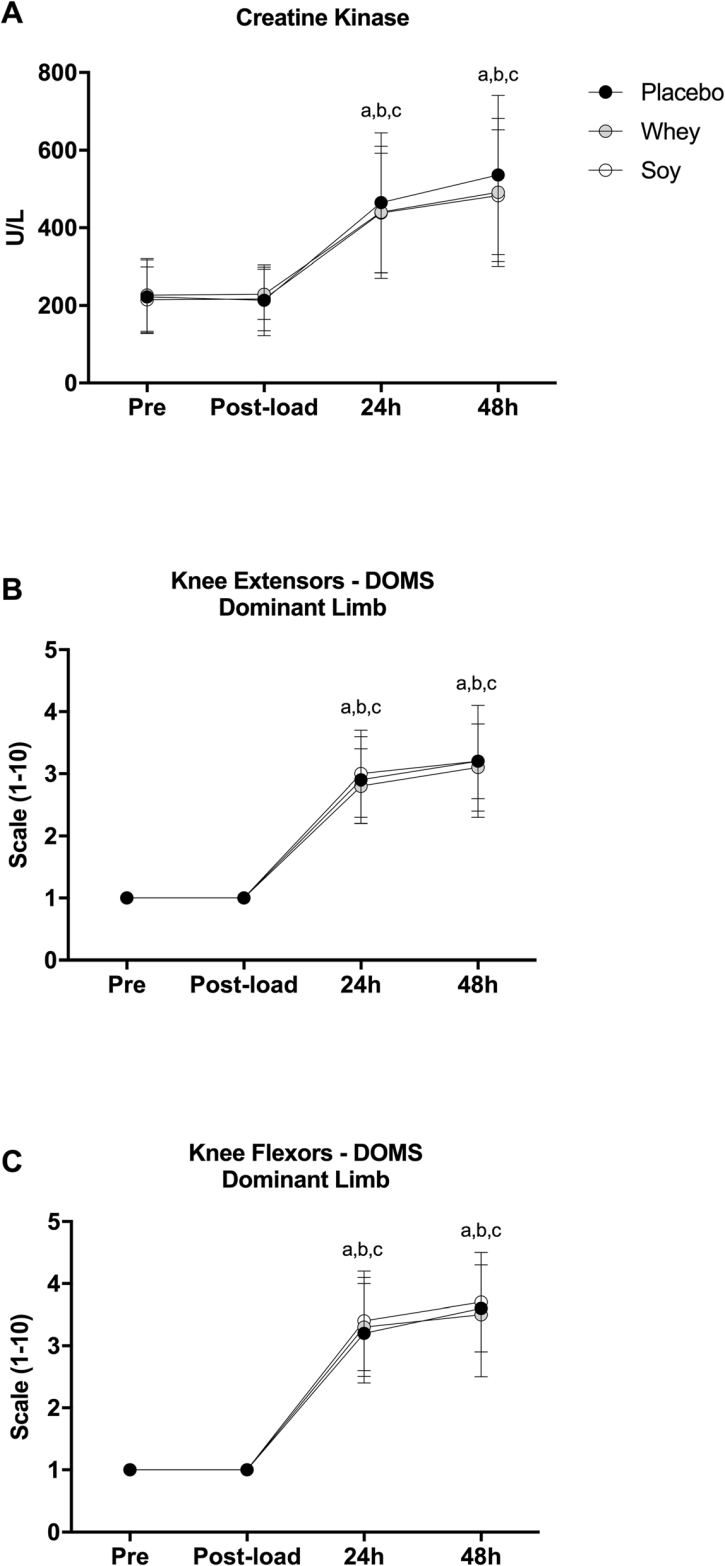
Changes in creatine kinase (CK) activity (**a**) and delayed onset of muscle soreness (DOMS) of the knee extensors (**b**) and knee flexors (**c**) of the DL. ^a^ denotes a significant difference with pre in placebo trial at *P* < 0.05. ^b^ denotes a significant difference with pre in whey trial at *P* < 0.05. ^c^ denotes a significant difference with pre in soy trial at *P* < 0.05

Creatine kinase demonstrated an almost 2-fold increase (compared to pre) in all trials at 24 h after speed-endurance training 1 (*P* < 0.001) and remained elevated until 48 h of recovery (*P* < 0.001), without any group differences (Fig. 3). Likewise, delayed-onset of muscle soreness of knee extensors and knee flexors of the dominant limb displayed an almost 3-fold rise (compared to baseline) in all trials both at 24 h (*P* < 0.001) and 48 h (*P* < 0.001) without any difference among trials (Fig. 3).

**Fig. 4:**
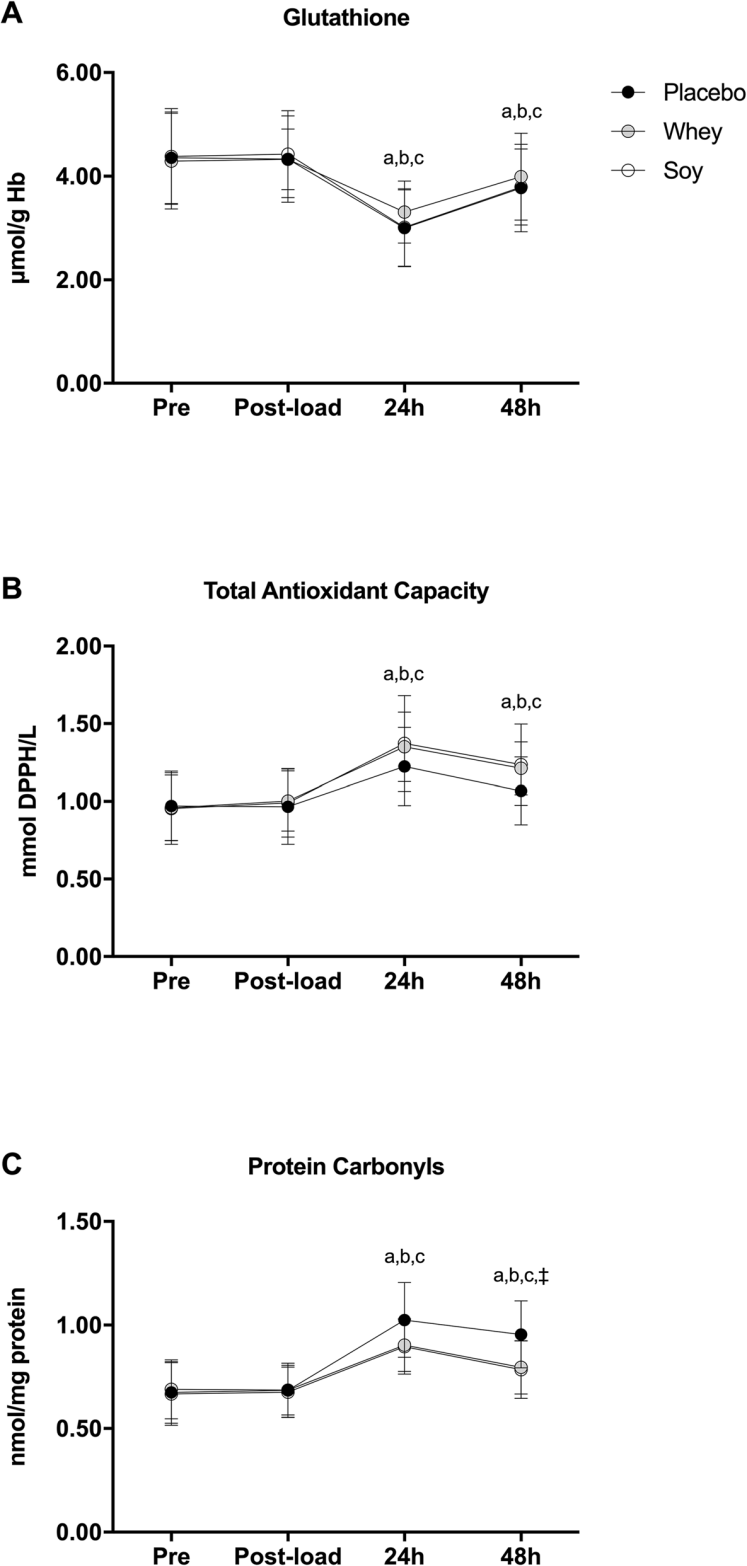
Changes in reduced glutathione (**a**), total antioxidant capacity (**b**) and protein carbonyls (**c**) levels. ^a^ denotes a significant difference with pre in placebo trial at *P* < 0.05. ^b^ denotes a significant difference with pre in whey trial at *P* < 0.05. ^c^ denotes a significant difference with pre in soy trial at *P* < 0.05. ^‡^ denotes a significant difference between soy and placebo trial within time-point at *P* < 0.05

Glutathione decreased similarly in all trials at 24 h (PL: -31%, *P* < 0.001; WP: -23%, *P* < 0.001; SP: -31%, *P* < 0.001) and 48 h (PL: -14%, *P* < 0.001; WP: -7%, *P* = 0.001; SP: -13%, *P* < 0.001) after speed-endurance training 1, compared to baseline (Fig. 4). Total antioxidant capacity and protein carbonyls increased in all trials at 24 h (total antioxidant capacity: PL: + 26%, *P* < 0.05; WP: + 41%, *P* < 0.001; SP: + 44%, *P* < 0.001 / Protein carbonyls: PL: + 52%, *P* < 0.001; WP: + 31%, *P* < 0.001; SP: + 34%, *P* < 0.001) and remained elevated until 48 h (total antioxidant capacity: PL: + 10%, *P* < 0.001; WP: + 26%, *P* < 0.001; SP: + 29%, *P* < 0.001 / Protein carbonyls: PL: + 42%, *P* < 0.001; WP: + 15%, *P* < 0.001; SP: + 18%, *P* < 0.001) after speed-endurance training 1 compared to baseline (Fig. 4). No trial-related differences were observed for total antioxidant capacity, whereas the rise in protein carbonyls at 48 h after speed-endurance training 1 was lower in SP (*P* = 0.04) and tended to be lower in WP (*P* = 0.061) as compared to PL (Fig. 4).

## Discussion

Τhis study is the first to investigate the effectiveness of WP and SP supplementation on performance recovery kinetics, exercise-induced muscle damage, and oxidative stress following speed-endurance training. Our findings suggest that supplementation with either whey or soy protein (reaching a total protein intake of 1.5 g protein/kg/day) may facilitate performance recovery following a speed-endurance training session, by enhancing the players’ ability to perform high-intensity running and high-speed running during a second speed-endurance training session performed 48 h following the first one.

The two speed-endurance training sessions applied in this study, were very intense with peak heart rate (~ 95% of maximum) and speed (~ 100% of maximum) reaching very close to their respective maximum values, while total distance and number of accelerations and decelerations performed were similar to those previously reported for this type of training [6]. The glycolytic metabolism was highly taxed as evidenced by the pronounced rise in blood lactate following speed-endurance training sessions, which is in line with previous reports [3, 5, 6].

A considerable decline in field performance was observed during the second speed-endurance training compared to the first one, corroborating previous findings suggesting that speed-endurance training sessions should be placed at least 72 h apart to allow players’ complete recovery [6]. Interestingly, both WP and SP supplementation mitigated this decline by enabling players to perform more HIR and HSR during the second speed-endurance training session, suggesting that increased dairy- and plant-based protein consumption may be a beneficial strategy during a congested soccer training schedule. Similar findings were previously reported for protein supplementation (80% casein, 20% whey) during a congested microcycle with two soccer matches performed 3 days apart [17], while co-ingestion of carbohydrates and protein has been shown to improve running performance and attenuate the decline of maximum speed [23]. This protein-mediated effect might be partly explained by a potential reduction of muscle fatigue following speed-endurance training. Although, we do not provide here any measures related to muscle fatigue, previous research revealed that protein supplementation, particularly that of fast digestible type, results in notable reduction of muscle fatigue during and following a 10-week, intense resistance training [24]. On the other hand, protein ingestion may favor skeletal muscle healing following exercise-induced muscle damage through stimulation of muscle protein synthesis and satellite cells activation and preservation of proteasome activity [19, 25, 26]. Therefore, the smaller reduction observed in high-intensity running and high-speed running during the second speed-endurance training session might be attributed on the protein-driven acceleration of the skeletal muscle healing process. Future studies though, applying muscle biopsies and advanced molecular techniques, including measurement of signaling proteins, satellite cells activation-related indices and proteasome function, are required to elucidate this scenario.

Speed-endurance training resulted in exercise-induced muscle damage induction, as indicated by the rise in delayed-onset of muscle soreness and creatine kinase that was of similar magnitude and duration with that reported previously [6]. However, exercise-induced muscle damage related markers remained unaffected by WP and SP supplementation. Previous investigations on the effect of protein supplementation on exercise-induced muscle damage markers have provided ambiguous results. For instance, in untrained males, WP isolate supplementation (1.5 g/kg/day) for 14 days following eccentrically-induced muscle damage failed to attenuate the rise in creatine kinase compared to placebo [10]. Likewise, when trained individuals were involved in a 7-day strenuous resistance training, WP supplementation did not affect serum creatine kinase [27]. In contrast, consumption of a whey-CHO blend attenuated the rise in creatine kinase during soccer training [28] while oat protein ingestion (25 g/day) mitigated the rise in delayed-onset of muscle soreness and creatine kinase before (14 days) and after (4 days) downhill running [29]. The discrepancy among studies, may be attributed to the large inter- and intra-individual variability as well as the clearance rate from the systemic lymphatic transport of creatine kinase [10, 30]. The exact mechanism linking protein ingestion with creatine kinase and delayed-onset of muscle soreness reduction following damaging exercise protocols is currently obscure. In this investigation, the protein-mediated preservation of high-intensity running and high-speed running during the second speed-endurance training session may be associated with its anti-inflammatory effects rather than an attenuation of exercise-induced muscle damage related markers [17].

Moreover, WP and SP supplementation did not have any impact on the recovery process in most of the indirect markers of exercise-induced muscle damage and performance examined. Specifically, concentric and eccentric isokinetic peak torque decreased at 24 h after speed-endurance training 1 similarly in all trials, as previously reported [6], without any effect of protein supplementation. In line with our observations, previous research revealed that WP hydrolysate and milk-based supplementation had no impact on isokinetic strength performance recovery following resistance exercise [31]. However, one study revealed that WP isolate (1.5 g/kg/day) attenuated the decline of isometric and isokinetic performance following exercise-induced muscle damage [10]. Although we failed to detect any statistically meaningful differences among trials in terms of maximal voluntary isometric contraction and isokinetic strength, a tendency (*P* = 0.06) for an attenuated decline in knee flexors eccentric strength of both limbs was noted in WP (~ 13%) and SP (~ 15%) compared to PL (~ 20%) after speed-endurance training 1. Moreover, in contrast to 30-m speed, repeated sprint ability and countermovement jump performance that was similarly deteriorated during recovery from speed-endurance training 1 in all trials, 10-m speed decreased in PL but not in WP and SP. Similarly, it has been reported that semi-skimmed milk consumption preserved 10-m sprint performance following damaging exercise but failed to enhance recovery of countermovement jump and 30-m sprint time [32], which is in coherence with comparable studies [8, 17]. Thus, it seems that protein supplementation has very little, if any, impact on indirect markers of performance recovery following speed-endurance training.

Exercise-induced muscle damage instigates an inflammatory response that provokes reactive oxygen species production mainly by migrating leukocytes through the NADPH-oxidase oxidative burst to remove cellular debris [33, 34]. Hydroxyl radical formation oxidizes proteins leading to the formation of protein carbonyls while a reduction in glutathione pool is indicative of increased intracellular glutathione consumption to counteract reactive oxygen species [33]. Increased evidence proposed an indirect antioxidant role for WP and SP supplementation [17, 19, 35], which is mainly attributed to their cysteine content that serves as a precursor molecule for intracellular glutathione synthesis and preservation of redox balance [35]. Furthermore, soy’s high content in isoflavones further fosters its role as an antioxidant agent [12]. However, in this study, we only observed an attenuated protein carbonyl rise at 48 h after speed-endurance training 1 in SP and a tendency (without reaching statistical significance) for an attenuated decline in glutathione and a more pronounced rise in total antioxidant capacity in WP. In contrast to our findings, it was previously shown that WP and SP supplementation prevented oxidative damage and decreased lipid peroxidation in exercising mice [12]. Furthermore, leucine-protein co-ingestion mitigated the superoxide release by neutrophils following intense exercise and up-regulated the glutathione stores in leukocytes [36]. An antioxidant role of protein supplementation during recovery from soccer match-play has been also observed [17], though it should be highlighted that the rise in protein carbonyls and the decline in glutathione levels observed at 24 h following the soccer match [17] were of smaller magnitude compared the respective changes induced by speed-endurance training in the present study.

A limitation in the current study, that should be highlighted, is the fact that our participants’ total energy, carbohydrate and protein intake were considerably lower than the relative amounts recommended for soccer players during in-season [15]. Particularly protein intake, that was set to 0.8–1 g/kg/day throughout the PL trial and during the adaptive period in WP and SP trials, was much below the 1.6–2.2 g of protein/kg day recommended for players during a typical in-season training microcycle (i.e. one training week) [15]. Thus, one might admit that the differences observed between trials are attributed to the low protein intake in PL trial and not to increased protein consumption applied in WP and SP trials. However, participants’ daily energy expenditure and metabolic demands were very low during the course of each trial, as they only participated in very light load training (3–40 min per session), including two speed-endurance training sessions in which the net working time was 4 min, and abstained from any type of moderate-to-vigorous physical activity. One should take into consideration that our experimental trials focused on speed-endurance training recovery and not the overall training volume of a pre- or an in-season training microcycle.

## Conclusions

In conclusion, increasing daily protein intake to 1.5 g/kg/day through either whey or soy protein supplementation facilitates recovery of field performance following speed-endurance training, in soccer players. Moreover, this effect was not related to an attenuation of exercise-induced muscle damage indices or oxidative stress. Given that WP and SP ingestion were equally effective, we propose that SP consumption may represent an efficient and cost-effective alternative to dairy proteins in respect to recovery from soccer-specific, high-intensity training, such as speed-endurance training.

## Supplementary Information

**Additional file 1. **The CONSORT Flow Diagram of the study.

**Additional file 2. ***P* value, effect size and confidence interval for all significant differences observed.

**Additional file 3.** Field activity and internal load responses during Speed-Endurance Training

**Additional file 4. **Changes in maximal voluntary isometric contraction.

## Data Availability

The datasets used and/or analyzed during the current study are available from the corresponding author on reasonable request.

## Abbreviations

WP: Whey protein
SP: Soy protein
PL: Placebo
