# Supplementary figures and images for: Effect of whey vs. soy protein supplementation on recovery kinetics following speed endurance training in competitive male soccer players: a randomized controlled trial
Source: J Int Soc Sports Nutr. 2021 Mar 16;18:23. doi: 10.1186/s12970-021-00420-w (PMC7968192; doi:10.1186/s12970-021-00420-w)

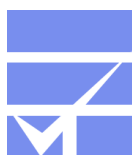

# CONSORT

TRANSPARENT REPORTING of TRIALS

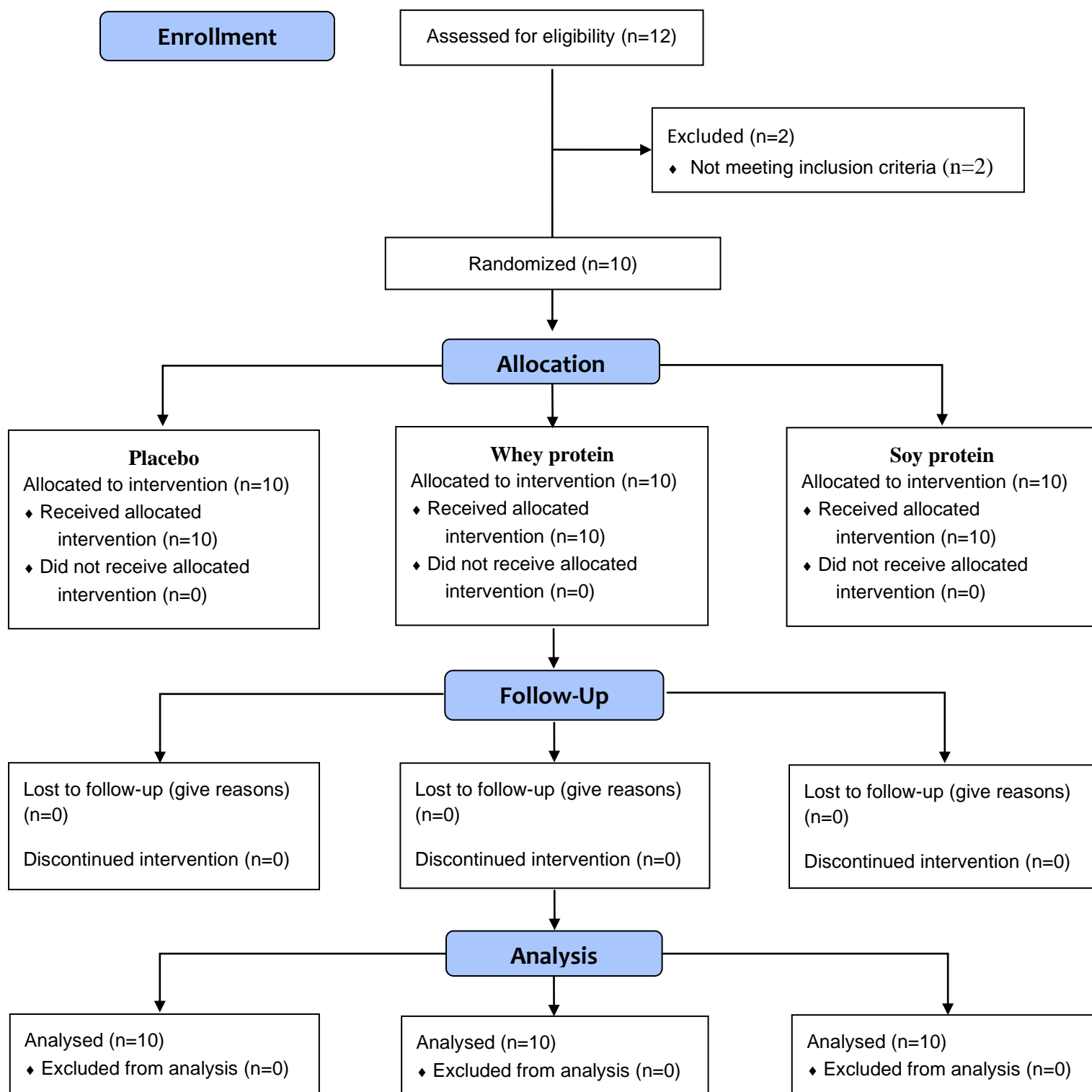

Supplement: Supplementary file 1 — Additional file 1. The CONSORT Flow Diagram of the study. [file 12970_2021_420_MOESM1_ESM.pdf]
