# Supplementary material for: Effect of whey vs. soy protein supplementation on recovery kinetics following speed endurance training in competitive male soccer players: a randomized controlled trial
Source: J Int Soc Sports Nutr. 2021 Mar 16;18:23. doi: 10.1186/s12970-021-00420-w (PMC7968192; doi:10.1186/s12970-021-00420-w)
Supplement: Supplementary file 2 — Additional file 2. P value, effect size and confidence interval for all significant differences observed. [file 12970_2021_420_MOESM2_ESM.docx]

| **Additional Table 2.** P value, effect size and confidence interval for all significant differences observed. | | |
| --- | --- | --- |
| **Variables** | **Time-dependent differences** | **Between-group differences** |
|  |  |  |
| **Field activity** |  |  |
| Maximum speed |  |  |
|  | PL, SEPT1 vs SEPT2: *P*<0.001/1.00/0.07;1.93 | WP vs PL: *P*=0.003/-1.48/-2.47; -0.49 |
|  | WP, SEPT1 vs SEPT2: *P*<0.001/0.82/-0.09;1.74  SP, SEPT1 vs SEPT2: *P*<0.001/0.83/0.08;1.74 | SP vs PL: *P*=0.011/-1.36/-2.33; -0.39 |
|  |  |  |
| Average speed |  |  |
|  | PL, SEPT1 vs SEPT2: *P*<0.001/3.75/2.30;5.21 | WP vs PL: *P*=0.019/-1.33/-2.30; -0.36 |
|  | WP, SEPT1 vs SEPT2: *P*<0.001/2.27/1.15;3.40  SP, SEPT1 vs SEPT2: *P*<0.001/2.62/1.43;3.82 |  |
|  |  |  |
| Average speed fatigue index |  |  |
|  | PL, SEPT1 vs SEPT2: *P*<0.001/-0.40/-1.29;0.48 |  |
|  | WP, SEPT1 vs SEPT2: *P*<0.001/-0.35/-1.24;0.53  SP, SEPT1 vs SEPT2: *P*<0.001/-0.37/-1.26;0.51 |  |
|  |  |  |
| High intensity running |  |  |
|  | PL, SEPT1 vs SEPT2: *P*<0.001/1.94/0.88;3.01 | WP vs PL: *P*<0.001/-2.86/-4.11; -1.62 |
|  | WP, SEPT1 vs SEPT2: *P*<0.001/1.43/0.45;2.42  SP, SEPT1 vs SEPT2: *P*<0.001/-2.06/0.97;3.14 | SP vs PL: *P*<0.001/-3.49/-4.88; -2.10 |
|  |  |  |
| High speed running |  |  |
|  | PL, SEPT1 vs SEPT2: *P*<0.001/0.96/0.04;1.89 | WP vs PL: *P*=0.015/-1.11/-2.05;0.17 |
|  | WP, SEPT1 vs SEPT2: *P*<0.001/0.61/-0.29;1.51  SP, SEPT1 vs SEPT2: *P*<0.001/0.65/-0.25;1.55 | SP vs PL: *P*=0.006/-1.99/-3.07; -0.92 |
|  |  |  |
| Intense accelerations |  |  |
|  | PL, SEPT1 vs SEPT2: *P*<0.001/1.07/0.14;2.01 |  |
|  | WP, SEPT1 vs SEPT2: *P*=0.003/0.71/-0.19;1.62  SP, SEPT1 vs SEPT2: *P*=0.001/0.94/0.02;1.87 |  |
|  |  |  |
| Intense decelerations |  |  |
|  | PL, SEPT1 vs SEPT2: *P*=0.001/0.39/-0.49;1.28 |  |
|  |  |  |
| Average heart rate |  |  |
|  | PL, SEPT1 vs SEPT2: *P*=0.001/0.82/-0.09;1.73 |  |
|  | WP, SEPT1 vs SEPT2: *P*=0.017/0.47/-0.42;1.36  SP, SEPT1 vs SEPT2: *P*=0.010/0.44/-0.45;1.33 |  |
|  |  |  |
| Blood lactate |  |  |
|  | PL, SEPT1: *P*<0.001/-9.91/-13.10; -6.71, SEPT2: *P*<0.001/-9.37/-12.40; -6.33 |  |
|  | WP, SEPT1: *P*<0.001/-10.47/-13.82; -7.11, SEPT2: *P*<0.001/-9.12/-12.08; -6.16  SP, SEPT1: *P*=0.010/-14.82/-19.50; -10.15, SEPT2: *P*=0.001/-14.52/-19.10; -9.94 |  |
|  |  |  |
| **Performance** |  |  |
| Concentric strength, knee extensors, dominant limb | | |
|  | PL, 24h: *P*<0.001/1.39/0.42;2.37 |  |
|  | WP, 24h: *P*<0.001/0.82/-0.10;1.73  SP, 24h: *P*<0.001/0.84/-0.07;1.76 |  |
|  |  |  |
| Concentric strength, knee extensors, non-dominant limb | | |
|  | PL, 24h: *P*<0.001/1.06/0.12;1.99 |  |
|  | WP, 24h: *P*<0.001/0.94/0.02;1.86  SP, 24h: *P*<0.001/0.99/0.06;1.92 |  |
|  |  |  |
| Concentric strength, knee flexors, dominant limb | | |
|  | PL, 24h: *P*<0.001/0.63/-0.27;1.53 |  |
|  | WP, 24h: *P*<0.001/0.62/-0.27;1.52  SP, 24h: *P*<0.001/0.59/-0.31;1.49 |  |
|  |  |  |
| Concentric strength, knee flexors, non-dominant limb | | |
|  | PL, 24h: *P*<0.001/0.56/-0.34;1.45 |  |
|  | WP, 24h: *P*<0.001/0.62/-0.28;1.52  SP, 24h: *P*<0.001/0.56/-0.33;1.46 |  |
|  |  |  |
| Eccentric strength, knee extensors, dominant limb | | |
|  | PL, 24h: *P*=0.005/0.60/-0.30;1.49 |  |
|  | WP, 24h: *P*=0.015/0.53/-0.36;1.42  SP, 24h: *P*=0.029/0.53/-0.36;1.42 |  |
|  |  |  |
| Eccentric strength, knee extensors, non-dominant limb | | |
|  | PL, 24h: *P*<0.001/0.91/-0.01;1.83 |  |
|  | WP, 24h: *P*<0.001/0.72/-0.18;1.63  SP, 24h: *P*<0.001/0.74/-0.16;1.65 |  |
|  |  |  |
| Eccentric strength, knee flexors, dominant limb | | |
|  | PL, 24h: *P*<0.001/1.19/0.24;2.14 |  |
|  | WP, 24h: *P*<0.001/1.02/0.09;1.95  SP, 24h: *P*<0.001/1.23/0.27;2.18 |  |
|  |  |  |
| Eccentric strength, knee flexors, non-dominant limb | | |
|  | PL, 24h: *P*<0.001/1.45/0.47;2.44 |  |
|  | WP, 24h: *P*<0.001/1.20/0.25;2.15  SP, 24h: *P*<0.001/1.13/0.18;2.07 |  |
|  |  |  |
| Maximal voluntary isometric contraction, knee extensors, non-dominant limb | | |
|  | PL, 1h: *P*<0.001/1.39/0.41;2.36, 2h: *P*=0.003/0.84/-0.08;1.75 |  |
|  | WP, 1h: *P*=0.001/1.24/0.29;2.20, 2h: *P*=0.005/0.80/-0.11;1.71  SP, 1h: *P*<0.001/1.35/0.38;2.32, 2h: *P*=0.005/0.79/-0.12;1.70 |  |
|  |  |  |
| 10-m speed |  |  |
|  | PL, 24h: *P*=0.036/-0.99/-1.92; -0.06 |  |
|  |  |  |
| 30-m speed |  |  |
|  | PL, 24h: *P*<0.001/-1.58/-2.59; -0.58 |  |
|  | WP, 24h: *P*<0.001/-0.81/-1.72;0.10  SP, 24h: *P*<0.001/-1.13/-2.08; -0.19 |  |
|  |  |  |
| Repeated sprint ability fatigue index | | |
|  | PL, 24h: *P*<0.001/-0.85/-1.77;0.07 |  |
|  | WP, 24h: *P*=0.004/-0.71/-1.61;0.20  SP, 24h: *P*=0.001/-1.09/-2.03; -0.15 |  |
|  |  |  |
| Countermovement jump |  |  |
|  | PL, 24h: *P*<0.001/0.88/-0.04;1.80 |  |
|  | WP, 24h: *P*=0.003/0.81/-0.10;1.72  SP, 24h: *P*=0.001/0.85/-0.06;1.77 |  |
|  |  |  |
| **Muscle damage** |  |  |
| Creatine kinase |  |  |
|  | PL, 24h: *P*<0.001/-1.60/-2.61; -0.59, 48h: *P*<0.001/-1.86/-2.91; -0.81 |  |
|  | WP, 24h: *P*<0.001/-1.48/-2.47; -0.49, 48h: *P*<0.001/-1.67/-2.69; -0.65  SP, 24h: *P*<0.001/-1.75/-2.78; -0.72, 48h: *P*<0.001/-1.78/-2.81; -0.74 |  |
|  |  |  |
| Delayed onset of muscle soreness, knee extensors, dominant limb | | |
|  | PL, 24h: *P*<0.001/-3.64/-5.07; -2.21, 48h: *P*<0.001/-3.28/-4.62; -1.94 |  |
|  | WP, 24h: *P*<0.001/-4.03/-5.55; -2.50, 48h: *P*<0.001/-4.03/-5.55; -2.50  SP, 24h: *P*<0.001/-4.07/-5.60; -2.53, 48h: *P*<0.001/-4.69/-6.38; -2.99 |  |
|  |  |  |
| Delayed onset of muscle soreness, knee flexors, dominant limb | | |
|  | PL, 24h: *P*<0.001/-3.79/-5.25; -2.32, 48h: *P*<0.001/-5.06/-6.85; -3.26 |  |
|  | WP, 24h: *P*<0.001/-3.76/-5.22; -2.31, 48h: *P*<0.001/-3.46/-4.84; -2.07  SP, 24h: *P*<0.001/-3.83/-5.31; -2.36, 48h: *P*<0.001/-4.42/-6.05; -2.79 |  |
|  |  |  |
| **Redox status** |  |  |
| Glutathione |  |  |
|  | PL, 24h: *P*<0.001/1.56/0.56;2.56, 48h: *P*<0.001/0.60/-0.30;1.49 |  |
|  | WP, 24h: *P*<0.001/0.77/-0.14;1.68, 48h: *P*=0.001/0.29/-0.59;1.17  SP, 24h: *P*<0.001/1.55/0.55;2.55, 48h: *P*<0.001/0.65/-0.25;1.54 |  |
|  |  |  |
| Total antioxidant capacity |  |  |
|  | PL, 24h: *P*=0.008/-1.07/-2.01; -0.13, 48h: *P*=.000/-0.45/-1.34;0.44 |  |
|  | WP, 24h: *P*<0.001/-1.76/-2.79; -0.73, 48h: *P*<0.001/-1.28/-2.24; -0.32  SP, 24h: *P*<0.001/-1.49/-2.48; -0.50, 48h: *P*<0.001/-1.08/-2.02; -0.14 |  |
|  |  |  |
| Protein carbonyls |  |  |
|  | PL, 24h: *P*<0.001/-2.06/-3.14; -0.98, 48h: *P*<0.001/-1.77/-2.80; -0.73 | SP vs PL, 48h: *P*=0.041/1.11/0.17;2.05 |
|  | WP, 24h: *P*<0.001/-1.60/-2.61; -0.59, 48h: *P*<0.001/-0.80/-1.71;0.11  SP, 24h: *P*<0.001/-1.56/-2.56; -0.55, 48h: *P*<0.001/-0.81/-1.72;0.10 |  |
|  |  |  |
| Abbreviations: PL, placebo; WP, whey protein; SP, soy protein; SEPT, speed-endurance production training. Note: Data are presented as p value/ES/95% CI. | | |
