# Supplementary material for: Effect of whey vs. soy protein supplementation on recovery kinetics following speed endurance training in competitive male soccer players: a randomized controlled trial
Source: J Int Soc Sports Nutr. 2021 Mar 16;18:23. doi: 10.1186/s12970-021-00420-w (PMC7968192; doi:10.1186/s12970-021-00420-w)
Supplement: Supplementary file 3 — Additional file 3. Field activity and internal load responses during Speed-Endurance Training [file 12970_2021_420_MOESM3_ESM.docx]

| **Additional Table 3.** Field Activity and Internal Load Responses During Speed-Endurance Training | | | | | | | | | |
| --- | --- | --- | --- | --- | --- | --- | --- | --- | --- |
|  | **Placebo** | | | **Whey** | | | **Soy** | | |
|  | **T1** | **T2** | **% Change** | **T1** | **T2** | **% Change** | **T1** | **T2** | **% Change** |
| Total Distance (m) | 133.9 ± 4.0 | 133.6 ± 3.9 | -0.2 ± 1.6 | 132.8 ± 4.5 | 134.4 ± 3.6 | 1.2 ± 2.1 | 133.0 ± 5.2 | 134.9 ± 3.9 | 1.5 ± 2.5 |
| Maximum Speed (km/h) | 33.2 ± 2.6 | 30.5 ± 2.5^*^ | -8.1 ± 1.7 | 33.4 ± 2.4 | 31.4 ± 2.2^*^ | -5.8 ± 1.2^†^ | 33.5 ± 2.5 | 31.4 ± 2.3^*^ | -6.1 ± 1.0^‡^ |
| Average Speed (km/h) | 19.2 ± 0.8 | 16.7 ± 0.4^*^ | -12.6 ± 4.7 | 19.3 ± 0.8 | 17.5 ± 0.7^*†^ | -9.5 ± 1.8 | 18.8 ± 0.7 | 17.0 ± 0.6^*^ | -9.9 ± 1.9 |
| Average Speed FI (%) | 10.2 ± 3.6 | 11.7 ± 3.5^*^ | 17.9 ± 12.2 | 9.9 ± 3.6 | 11.2 ± 3.4^*^ | 15.2 ± 8.7 | 10.6 ± 3.4 | 11.9 ± 3.2^*^ | 15.7 ± 14.2 |
| High-Intensity Running (m) | 117.0 ± 6.5 | 103.9 ± 6.3^*^ | -11.2 ± 1.0 | 116.2 ± 5.3 | 107.4 ± 6.3^*^ | -7.7 ± 1.3^†^ | 117.7 ± 4.3 | 108.6 ± 4.1^*^ | -7.7 ± 0.9^‡^ |
| High-Speed Running (m) | 40.2 ± 7.9 | 32.4 ± 7.5^*^ | -19.8 ± 3.0 | 39.7 ± 8.6 | 34.0 ± 9.1^*^ | -15.2 ± 4.7^†^ | 40.3 ± 9.1 | 34.4 ± 8.1^*^ | -14.6 ± 1.8^‡^ |
| Intense Acc counts (>2 m/s^2^) | 6.1 ± 1.2 | 4.9 ± 0.9^*^ | -18.5 ± 12.1 | 6.3 ± 1.2 | 5.4 ± 1.2^*^ | -14.2 ± 11.1 | 6.3 ± 1.1 | 5.3 ± 0.9^*^ | -14.6 ± 17.0 |
| Intense Dec counts (>2 m/s^2^) | 6.1 ± 1.1 | 5.7 ± 0.8^*^ | -5.8 ± 7.5 | 6.5 ± 1.0 | 6.3 ± 0.9 | -2.9 ± 6.0 | 6.4 ± 1.0 | 6.2 ± 0.8 | -2.7 ± 5.7 |
| Maximum HR (beats/min) | 182.9 ± 4.2 | 183.4 ± 4.1 | 0.3 ± 2.7 | 184.5 ± 4.0 | 183.4 ± 3.4 | -0.6 ± 1.2 | 184.5 ± 3.8 | 184.0 ± 3.6 | -0.3 ± 1.1 |
| Average HR (beats/min) | 159.0 ± 7.1 | 154.1 ± 3.7^*^ | -3.0 ± 2.9 | 157.4 ± 6.9 | 154.2 ± 5.9^*^ | -2.0 ± 2.0 | 159.5 ± 7.4 | 156.0 ± 7.7^*^ | -2.2 ± 2.4 |
| Energy Expenditure (kcals) | 107.2 ± 9.8 | 94 ± 7.7 | -12.3 ± 2.1 | 109.3 ± 9.7 | 98.1 ± 9.3 | -11.4 ± 2.4 | 105 ± 9.3 | 95.4 ± 8.7 | -9.1 ± 3.1 |
| T, training; FI, fatigue index; Acc, accelerations; Dec, decelerations; HR, heart rate. Note: Data represent the average values during the 8 repetitions of the speed-endurance training protocol and are presented as means ± SD. ^*^Significant difference from T1 within the same trial at *P* < .05. ^†^Significant difference between Whey and Placebo at *P* < .05. ^‡^Significant difference between Soy and Placebo at *P* < .05. | | | | | | | | | |
