# Supplementary material for: Effect of whey vs. soy protein supplementation on recovery kinetics following speed endurance training in competitive male soccer players: a randomized controlled trial
Source: J Int Soc Sports Nutr. 2021 Mar 16;18:23. doi: 10.1186/s12970-021-00420-w (PMC7968192; doi:10.1186/s12970-021-00420-w)
Supplement: Supplementary file 4 — Additional file 4. Changes in maximal voluntary isometric contraction. [file 12970_2021_420_MOESM4_ESM.docx]

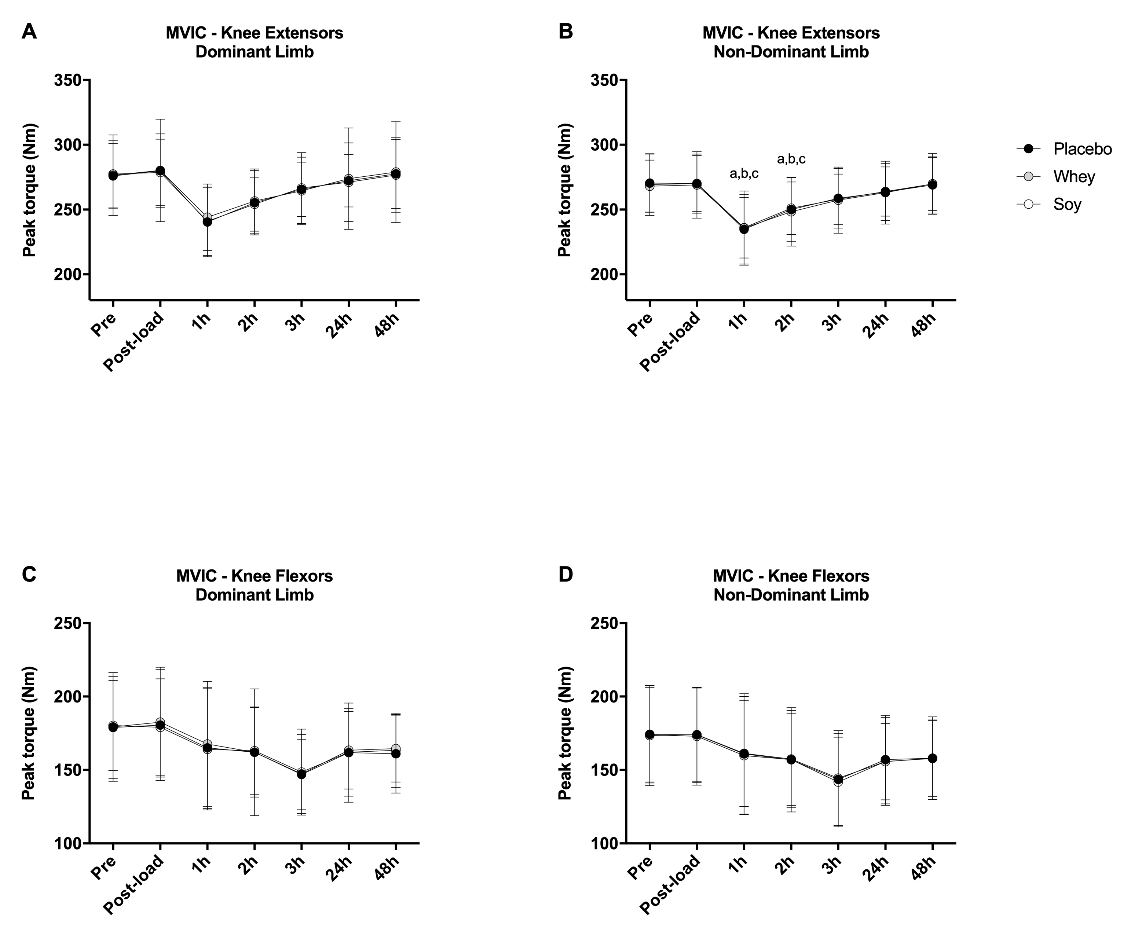


Changes in maximal voluntary isometric contraction (MVIC) in knee extensors of the dominant (A) and non-dominant limb (B), and in knee flexors of the dominant (C) and non-dominant (D) limb. ^a^ denotes a significant difference with pre in placebo at *P* < 0.05. ^b^ denotes a significant difference with pre in whey at *P* < 0.05. ^c^ denotes a significant difference with pre in soy at *P* < 0.05.
